# Evaluating working memory in young individuals with normal hearing through tele-assessment and traditional assessment: a comparative study

**DOI:** 10.3389/fdgth.2025.1499737

**Published:** 2025-02-17

**Authors:** Kavassery Venkateswaran Nisha, N. Devi, B. Vandana, E. Rashmi, Shraddha A. Shende, Raksha A. Mudar

**Affiliations:** ^1^ Center for Hearing Sciences, Department of Audiology, All India Institute of Speech and Hearing (AIISH), Mysuru, India; ^2^ Department of Audiology, All India Institute of Speech and Hearing, Mysuru, India; ^3^ Department of Communication Sciences and Disorders, Illinois State University, Normal, IL, United States; ^4^ Department of Speech and Hearing Science, University of Illinois Urbana-Champaign, Champaign, IL, United States

**Keywords:** tele-assessment, cognition, working memory, young adults, face-to-face, workload

## Abstract

**Aim:**

This pilot study examined the feasibility of tele-assessment of working memory (WM) compared to conventional face-to-face assessment.

**Methods:**

In total, 15 young adults aged between 18 and 30 years who were native speakers of Kannada with normal hearing completed three WM tests in Indian English: forward digit span, backward digit span, and n-back task through tele-assessment and in-person/face-to-face assessment. The National Aeronautics and Space Administration (NASA) task load index, which assesses subjective workload, was used to determine the difficulties across the two modes of assessment.

**Results:**

Paired comparison *t*-tests showed no significant differences in performance in the forward digit span (*p* = 0.29), backward digit span (*p* = 0.71), and n-back (*p* = 0.66) tasks across the two assessment conditions. Furthermore, the NASA task load index did not differ across the two assessment conditions for forward digit span (*p* = 0.29), backward digit span (*p* = 0.71), and n-back (*p* = 0.66). The Wilcoxon signed-rank test showed that the backward digit span task was the most difficult among the working memory tasks in both modes of assessment. The findings of our pilot study suggest that both modes can be used successfully to assess working memory, and tele-assessment yields similar results to face-to-face WM assessment in young normal-hearing adults. These results support the feasibility of conducting WM tests via tele-assessment, which has implications for use in clinical populations.

## 1 Introduction

Working memory (WM) is the ability of the brain to temporarily store and manipulate information necessary to execute complex cognitive tasks (1). WM plays a vital role in day-to-day listening, including the processing of spoken language, which is crucial for speech comprehension, especially in adverse listening conditions. A growing body of evidence suggests that WM is essential for facilitating speech perception in noise (SIN) even in the absence of hearing loss (2). The Ease of Language Understanding (ELU) model explains the role of WM in language understanding across a wide range of conditions, including normal or impaired hearing, uni-modal and bi-modal language inputs, verbal and sign language, and varying environmental challenges (3). This model also explains the interaction of WM with long-term memory (LTM). According to this model, multimodal speech information is Multimodally Bound into PHOnological representations in an episodic buffer called RAMBPHO. The matching between the RAMBPHO-delivered sub-lexical information and the phonological representation in the semantic LTM is critical for lexical retrieval and involves rapid top-down processing. Explicit and deliberate WM processes such as inference-making, semantic integration, switching of attention, storing of information, and inhibiting irrelevant information are critical to reconciling mismatches between RAMBPHO output and LTM representations. Other than top-down processing linked to WM, other explicit factors such as background noise, the listener's hearing abilities or impairments, and any signal processing performed by hearing assistance devices all play crucial roles in how effectively speech is perceived and understood. The relative contributions of explicit and implicit processes continuously fluctuate during SIN (3).

Empirical evidence supports the link between WM and “SIN” at word and sentence levels at varying signal-to-noise ratios in numerous studies (4–6). Lad et al. (4) found that the SIN performance of younger listeners is predicted by phonological WM capacity, which relates to an individual's ability to recognize and distinguish different frequency speech sounds. Füllgrabe and Rosen (5) extended these findings to middle-aged and older adults, in whom they reported a significant correlation between SIN scores and WM ability. Vermeire et al. (6) also reported that older adults had a significantly lower WM capacity and greater difficulty in understanding sentences in noise than younger adults. Similarly, Gordon-Salant and Cole (7) reported that adult listeners with a low WM capacity had difficulty understanding speech because of background noise, whereas listeners with a higher WM capacity easily capitalized on the contextual cues and performed significantly better. In summary, WM is crucial for understanding speech in the presence of noise in normal-hearing adults. To better support the needs of individuals with hearing loss, it is important for audiologists to establish ways to assess WM in the clients they serve.

WM is traditionally assessed face-to-face using tasks such as the digit span and n-back tasks. In the cognitive literature, the forward and backward digit span tasks are not considered a direct measure of WM (8), however, a few researchers have hinted that the tasks involve both attention and memory and should be regarded as a part of WM assessment (9). In the forward span task, participants must recall a gradually increasing sequence of auditory stimuli in the exact order presented by tapping into their attentional mechanisms. In the backward span test, the ability of the participant to recall the stimuli in reverse order is tested (10). The n-back task is a complex task that includes the encoding of stimuli and the monitoring, storage, and continuous maintenance of information prior to recall (11). A series of digits is presented and the n-back task requires the participant to match the stimulus to the one that occurred “n” positions back in the sequence. The n-back task includes different conditions such as “0-back,” “1-back,” “2-back,” and “3-back.”

During the COVID-19 pandemic, in-person cognitive assessments in regular healthcare practices were limited, highlighting the need to develop alternate options, such as those leveraging technology (12). Technological advancements, including videoconference-based assessment methods (audio–video platforms), have become more widespread (12), making tele-assessments in hearing easily accessible. Tele-assessments can potentially overcome general obstacles including long-distance travel to hospitals, adverse weather conditions, and limited availability of healthcare professionals (13). Indeed, many investigations have reported the utility of tele-assessment as an alternative option for in-person face-to-face assessment of cognitive function. These tele-assessments have been found to be valid and reliable for both urban and rural populations. The potential benefits of telehealth are especially alluring in the area of global hearing healthcare globally which has seen a shortage of healthcare experts who can offer audiological services to the rising number of people in need of treatment (13). This is particularly relevant for under-resourced regions in developing countries such as India.

Despite the expansion of telehealth services, information related to the reliability of cognitive tele-based assessments is still emerging. Out of the limited studies available, a study by Rivella et al. (14) reported the validity of tele-assessment of executive functions (TeleFE tool) and its potential in clinical practice in children. The TeleFE tool was a web-based platform for teachers/parents to evaluate a child’s inhibition (Go/NoGo), updating (N-back), flexibility (Flanker task), and planning (daily planning task). Stolwyk et al. (15) reported the feasibility, acceptability, and alternative measurement of in-person assessment in young onset dementia. The results of the study showed no significant differences between in-person and tele-neuropsychology assessment for the revised Hopkins Verbal Learning Task, Mini-Mental Status Examination, and Oral Symbol Digits Modalities Test. Hernandez et al. (12) demonstrated remote cognitive assessments had good reliability using a battery of cognitive tests. There was a high correlation (*r* = 0.90) of tele-assessment with face-to-face administration. In normal-hearing individuals aged 6 to 14 years, the utility of a web-based platform for remote assessment using cognitive tests (GO/NOGO, Flanker test, n-back task, and executive function questionnaires) in children and adolescents has been documented (16). Extending the application of tele-assessment in cognition to healthy older adults, Cyr et al. (17) compared cognitive assessments carried out at home vs. in the laboratory and reported no significant effect on test conditions (18). While great strides have been made in examining the utility of tele-assessment of cognition, information in the Indian context remains limited. Understanding how well WM tasks can be assessed remotely is important. This knowledge can help us better utilize available resources and services to measure cognitive ability in remote locations.

Studies have also revealed barriers in addition to facilitators in the use of tele-assessments. For instance, Aguilar and Leguizamón (19) reported the advantages and disadvantages of virtual and face-to-face neuropsychological test assessments based on a survey among 20 healthy young adults. They stated that the participants had better flexibility and comfort with limited travel time and cost in the tele-sessions than in the face-to-face sessions; however, they lacked in-person interactive time and direct eye contact with the examiner in the tele-mode. Another research study reported that some participants were concerned about perceiving visual stimuli and hearing auditory stimuli during online assessment due to sensory reasons (20). Considering these issues, a good first step when examining the feasibility of tele-assessments, including those for WM, is to test their utility in participants with normal hearing and vision, which was done in the current study (see details in the Methods section).

The development of tele-assessment of WM and examining its applicability in a variety of populations is crucial for resolving the inconsistencies in the literature. Furthermore, it is critical to compare how these tele-assessments compare to face-to-face assessments before broader community-based implementation is attempted and examine differences in participants’ perceived workload in different administration modalities. In addition, to understand the feasibility of using tele-assessment of WM, it is crucial to examine whether there are differences in complexity and mental load observed across WM tasks compared to the conventional face-to-face assessment. This pilot study assessed and compared WM examined through tele-mode and face-to-face assessment in an adult population aged 18 to 30 years with normal hearing and normal vision in Mysore, an urban region in southern India.

## 2 Methods

### 2.1 Participants

This preliminary study was conducted on 15 young individuals aged 18–30 years (mean = 25.4, SD = 3.2). The total number of male (mean age = 24; SD = 1.5) and female participants (mean age = 25; SD = 3.5) was 5 and 11, respectively. All the participants were native Kannada speakers from Mysore (Karnataka state), a region in southern India, and had a basic education (10th grade) with good proficiency in the English language. They could read and write in both Kannada and English. All the participants passed the neuropsychological evaluation screening tool (NEST) with no reported history of neurological impairment or psychological conditions such as attention deficit hyperactivity disorder, schizophrenia, depression, or emotional trauma that could impact cognition. The present study was approved by the institutional ethics committee at the All India Institute of Speech and Hearing, Mysore, India (Ref. no.: SH/ARF-FC/AUD-5/2022–23, dated 28 September 2022), and the ethical guidelines for bio-behavioral research (21) were followed. Written informed consent from the participants was received before the commencement of the study procedures.

Each participant underwent a detailed audiological assessment consisting of otoscopy, pure tone audiometry, otoacoustic emission, tympanometry, and acoustic reflex thresholds. The audiological evaluation was completed by an audiologist in a sound-treated room with permissible ambient noise limits as per ANSI S3.1, 1999. All the participants had hearing sensitivity within normal limits with air conduction (AC) and bone conduction (BC) thresholds within 15 dB hearing level (HL) between the frequency range of 250 Hz and 8 kHz measured using a bracketing procedure using a Grason-Stadler (GSI-61) audiometer under TDH-39 supra-aural headphones (28). Participants with no history of hearing loss, head trauma, or usage of ototoxic drugs were included in the test. All the participants had normal middle ear status with a type “A” tympanogram measured using a GSI-Tympstar middle ear analyzer. All the participants had normal ipsilateral and contralateral acoustic reflexes. Otoacoustic emissions were recorded using otodynamics instrument [Institute of Laryngology and Otology (ILO)] version 6 at two pure tones of frequencies “f1” and “f2” tested at two constant intensities with a constant f2/f1(1:2) ratio. Otoacoustic emissions (OAEs) obtained above the noise floor were considered to measure normal outer hair cell functioning. WM was assessed in a quiet room with minimal audio–visual distractions. The procedure included an assessment of WM using the forward digit span, backward digit span, and n-back tasks.

### 2.2 Working memory assessment

The tests were performed in tele-mode and face-to-face mode with the order randomized across participants.

#### 2.2.1 Conventional face-to-face mode of assessment

**Figure 1:**
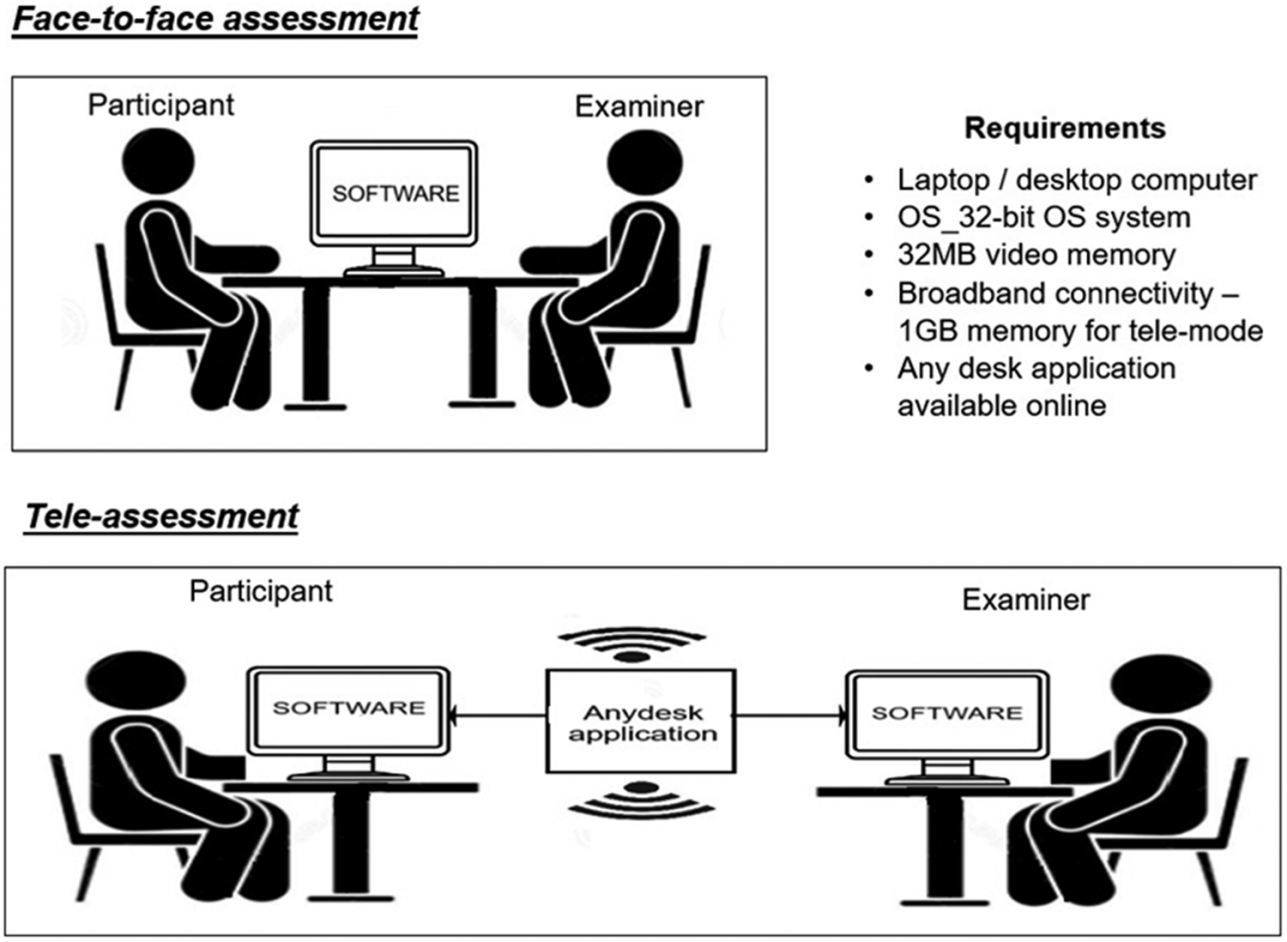
Schematic diagram of the test procedures in the two modes of assessment.

The cognitive tasks were carried out using the examiner's laptop (Intel® Core™ i3-4005U CPU @ 1.70 GHz). Stimuli for the WM tasks were presented through an auditory-based cognitive software, “Smriti Shravan,” that consists of paradigms to evaluate WM skills and speech perception in noise. The software includes an auditory presentation of the stimuli and a written response screen for participants to type their respective responses, which were documented for each participant. The test stimuli were presented at the intensity of 65 dB sound pressure level (SPL), calibrated using a sound level meter (SLM) in the ears of the Knowles Electronics Manikin for Acoustic Research (KEMAR). Testing was carried out without an Internet connection and responses were recorded in the software (Figure 1).

#### 2.2.2 Tele-assessment

The tele-assessment used two laptops, one for the examiner and another for the participant. Technical specifications for the laptops include a 32-bit operating system (OS), a dual-core processor with 32 MB of video memory, 1 GB of memory, and broadband connectivity**.** Participants were asked to remotely connect to the examiner's laptop through an application called “AnyDesk.” The “Smriti Shravan” software on the examiner's system monitored the participant's responses online using the Internet. For the tele-assessment, the participants were seated in a soundproof room with a laptop that was remotely connected to the examiner's laptop through the “Any Desk” application. The laptop was placed in front of them. Only the examiner had access to the software, including the administration of the test on the participant's computer. The tele-assessments and conventional assessments were conducted within 3–5 days of each other, with the order counterbalanced across all participants (Figure 1). Both assessments were carried out by an audiologist at the institute’s campus. The audiologist had a master's degree in Audiology, two years of post graduate experience and a valid professional registration number (from Rehabilitation council of India), which sufficed the legal requirements for practicing clinical audiology within India.

### 2.3 Test procedure

The WM tests in Indian English were assessed via in-person visits and tele-mode for all the participants. The tasks included the forward digit span, backward digit span, and n-back tasks. The test stimuli were presented using Sennheiser HD 449 circumaural headphones at 65 dB SPL. The National Aeronautics and Space Administration (NASA) workload questionnaire was administered to record the workload of the participants after completion of each mode of assessment, i.e., once after the tele-assessment and once after the conventional assessment. Following the administration of the workload questionnaire, an informal interview comprising aspects related to technology, namely comfort with technology, audibility, and level of satisfaction, was conducted at the end of each assessment condition for all the participants.

#### 2.3.1 Working memory tasks

##### 2.3.1.1 Forward and backward digit span tasks

**Figure 2:**
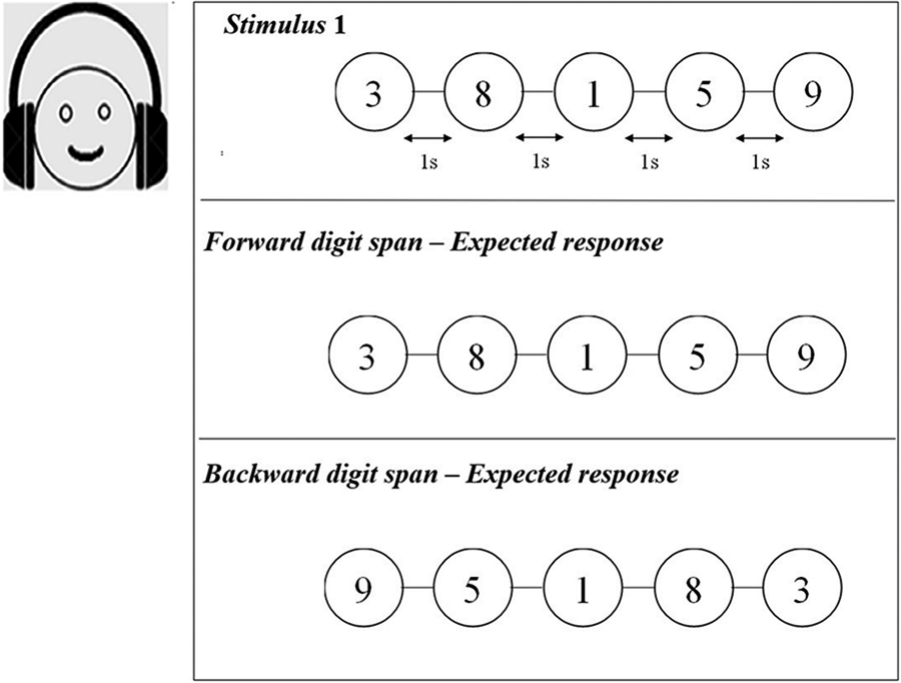
Schematic representation of forward and backward digit span tasks. Auditory digits were presented with an inter-stimulus interval of 1 s.

A sequence of digits (numbers from 1 to 9) was presented in English binaurally through Sennheiser headphones to the participants. The participants were instructed to respond by typing the digits in the same order presented in the forward digit span task and in the reverse order in the backward digit span task. For both the forward and backward digit span tasks, the duration of each digit in the sequence was 1,000 ms and there was an inter-stimulus interval (ISI) of 1,000 ms with a response time of 5,000 ms. All the participants in the study completed both the digit span tasks in the given response time window. The task level varied from level 1, a simple task including two digits, to level 8, which presented nine digits (Figure 2). The adaptive staircase procedure was incorporated to vary the level of difficulty by tracking the participant's performance. For example, for a three-digit recall task, if the participant recalled the digits correctly, the test then moved on to a four-digit recall. However, if a participant failed to recall the numbers, the task remained at the same level. The participants completed three practice tasks before each WM test. The presentation of stimuli in the sequence was randomized to avoid the practice effect. The responses for both tasks were obtained. The highest number of digits recalled in the tasks was considered the recall score for the forward and backward digit span tasks. The highest forward digit span score obtained in the conventional assessment was termed FsQ_C, while that in the tele-assessment it was designated FsQ_T. Similarly, for the backward digit span, the highest scores were designated BsQ_C and BsQ_T for the conventional and tele-assessments respectively.

##### 2.3.1.2 n-back task

**Figure 3:**
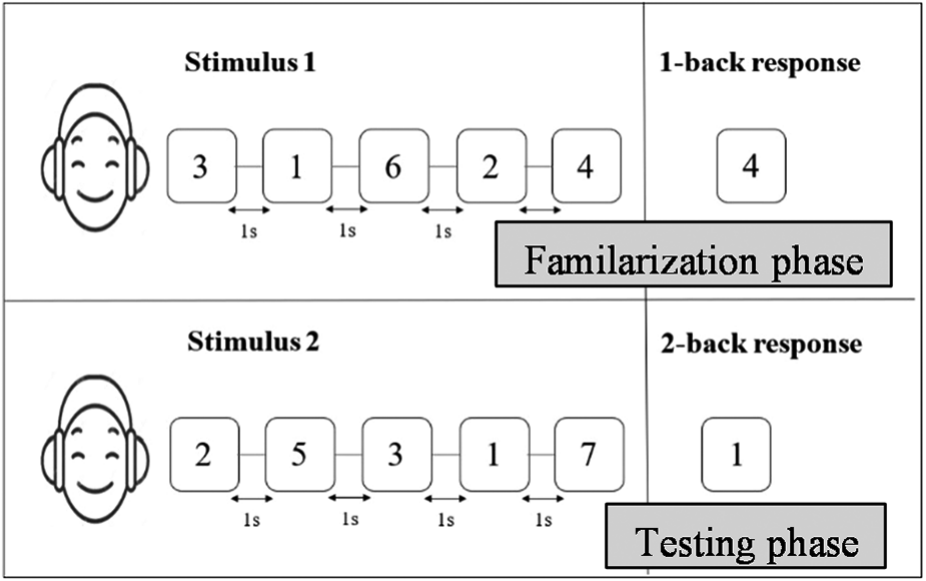
Schematic representation of the n-back task. Stimuli were presented with an inter-stimulus interval of 1 s.

In the n-back task, the participants were asked to match the presented stimulus with one that was presented “n” places back in a sequence. All the participants completed the 1-back and 2-back conditions, in which the participants had to listen to a string of audio digits and respond by recalling the last and second-last digits in the sequence, as shown in Figure 3. The length of the string of digits was varied from 4 to 10 with an ISI of 1,000 ms and a response time of 5,000 ms. The participants were instructed to carefully listen to the series of digits that were randomly presented, memorize the numbers, and after completion of the digit string, type in the last and second-last number from the series of numbers in the software for the 1-back and 2-back tasks respectively. The 1-back task was used in the practice tasks, and for analysis, the 2-back scores, which have a higher mental load, were used for comparison between the two conditions. The highest n-back scores obtained in the conventional mode of assessment were termed 2backQ_C, while in the tele-mode of assessment, it was designated 2backQ_T.

#### 2.3.2 Administration of the NASA workload index

The NASA task load index is a multidimensional rating scale (22) that has six domains including mental demand, physical demand, temporal demand, performance, effort, and frustration. The NASA task load index provided an overall workload score (for example, the amount of mental or physical effort they exerted) for all participants based on a weighted average of ratings on six subscales for each task, i.e., for the forward digit span (FsQ_C), backward digit span (BsQ_C), and n-back (2backQ_C) tasks in the conventional face-to-face assessment and in the tele-assessment (FsQ_T, BsQ_T, and 2back Q_T), respectively. The NASA workload index is scored from 0 to 100 with “0” being the lowest score indicating a reduced workload for a particular task and “100” being the highest score. Weighted scores were obtained by assigning percentages to each subscale based on their subjective judgment of its importance in which participants were asked to select the subscale that they found more burdening (causing a higher task load) compared to the others. The total workload was calculated by multiplying the ratings and weighted scores and dividing by 15, as recommended in the standard procedure (22). The workload scores obtained were utilized to compare the mental effort needed to complete the tasks in the two modes of WM assessment. The NASA questionnaire was administered after completion of all three tests in both the face-to-face and tele-assessments. We analyzed the participants’ ratings of each NASA subscale. For each subscale, we calculated how many participants reported significant difficulty (defined as ratings above 50% on a subscale). Taking the mental demand subscale (range: 0–100) as an example, in the tele-assessments, 8 out of 15 participants (53%) reported difficulty levels exceeding 50%. For the face-to-face assessment, 9 out of 15 participants (60%) reported high difficulty levels. These results are presented in Table 1.

**Table 1:** The number of times a participant rated each subscale as at least 50% difficulty across all WM tasks in the face-to-face and tele-assessments.

Subscales	Face-to-face assessment (%)	Tele-assessment (%)
Mental demand	53	60
Physical demand	33	26
Temporal demand	60	66
Effort	46	53
Performance	40	40
Frustration	33	40

### 2.4 Statistical analyses

The data were analyzed using Statistical Package for the Social Sciences (IBM SPSS 26 version, Chicago, IL, USA) software. The Shapiro–Wilk test of normality demonstrated a normal distribution for the data from the digit tasks (forward and backward). Thus, paired sample *t*-tests were used to examine the differences between the modes of administration. However, the data from the 2-back task was not normally distributed. Thus, the non-parametric equivalent Wilcoxon signed-rank test was used to compare the scores of this WM task from the two modes of assessment. Whenever significant differences were found, the effect size was calculated based on Cohen's d formula for *t*-tests (23), while the effect size for the non-parametric Wilcoxon test was calculated based on Rosenthal's formula (24). The Friedman test was utilized to compare the NASA workload task questionnaire scores that did not adhere to normality. This test was conducted to analyze how the workload differed between the various tasks (forward and backward digit tasks and the 2-back task) within each assessment mode. To examine the effect of the mode of assessment on mental load (NASA workload scores), the Wilcoxon sign test was conducted. The participants’ ratings of each subscale were utilized to analyze the number of times each subscale was rated as difficult and the data were subjected to a chi-square test for statistical analysis.

## 3 Results

### 3.1 Comparison of the WM scores of young normal-hearing individuals in the face-to-face and tele-assessments

**Figure 4:**
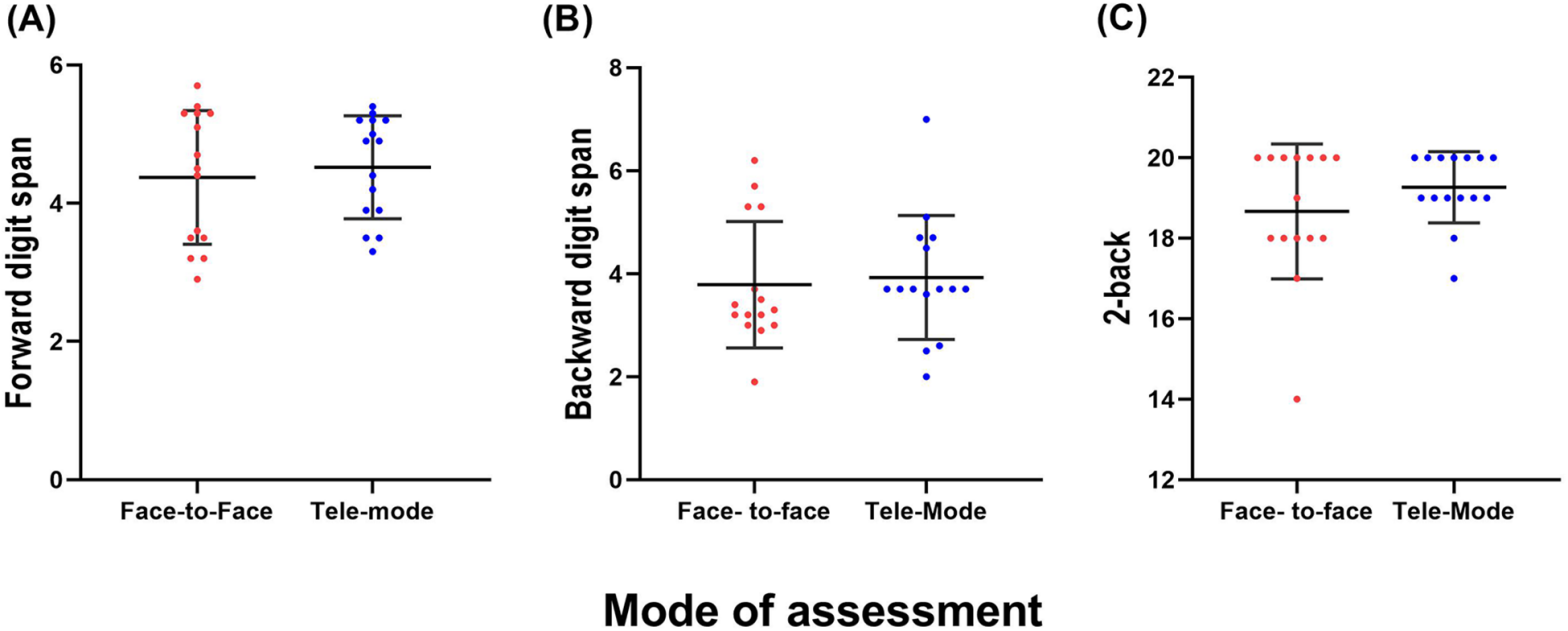
The mean scores of the **(A)** forward digit span, **(B)** backward digit span, and **(C)** n-back tasks in the face-to-face and tele-assessments. The error bars indicate one SD.

Descriptive statistics comprising means and standard deviations for the forward digit task, backward digit task, and n-back (2-back) task are depicted in Figure 4. The paired sample *t*-test revealed no significant differences in the scores obtained between the two modes of assessment for the forward digit span [*t* (14) = 1.45, *p* = 0.16] and backward digit span [*t* (14) = 0.193, *p* = 0.84] tasks. Similarly, the non-parametric analysis using the Wilcoxon signed-rank test showed no significant differences between the two modes of assessment in the 2-back scores (*p* = 0.08).

### 3.2 Comparison of the workload index ratings of conventional face-to-face mode of assessment and tele-mode of assessment for each WM task

Descriptive statistics comprising the overall workload scores from the questionnaire for each participant in the forward digit task, backward digit task, and 2-back task for both tele-mode and face-to-face mode of assessment are given in Table 2.

**Table 2:** The mean and standard deviation, and median and interquartile range of workload questionnaire scores for the three tasks in the conventional face-to-face and tele-assessments.

	Face-to-face assessment	Tele-assessment	Face-to-face assessment	Tele-assessment
	Mean ± SD	Mean ± SD	Median (IQR)	Median (IQR)
Forward span (FsQ)	55.73 ± 4.74	53.73 ± 3.67	56 ± 6	53 ± 3
Backward span (BsQ)	65.40 ± 5.34	62.47 ± 4.4	67 ± 7	62 ± 4
n-back (2backQ)	40.07 ± 5.62	41.87 ± 7.06	39 ± 14	47 ± 11

The results showed that the workload index scores for the forward digit span [*t* (14) = 1.09, *p* = 0.29] and backward digit span tasks [*t* (14) = 0.36, *p* = 0.71] in the tele and conventional face-to-face modes of assessment did not display any statistically significant differences. Further, the results of the non-parametric Wilcoxon signed-rank test for the workload index for the 2-back task in the two modes of assessment also revealed no significant differences (/Z/ = 1.316; *p* = 0.66).

### 3.3 Comparison of workload scores for WM tasks in each mode of assessment

**Figure 5:**
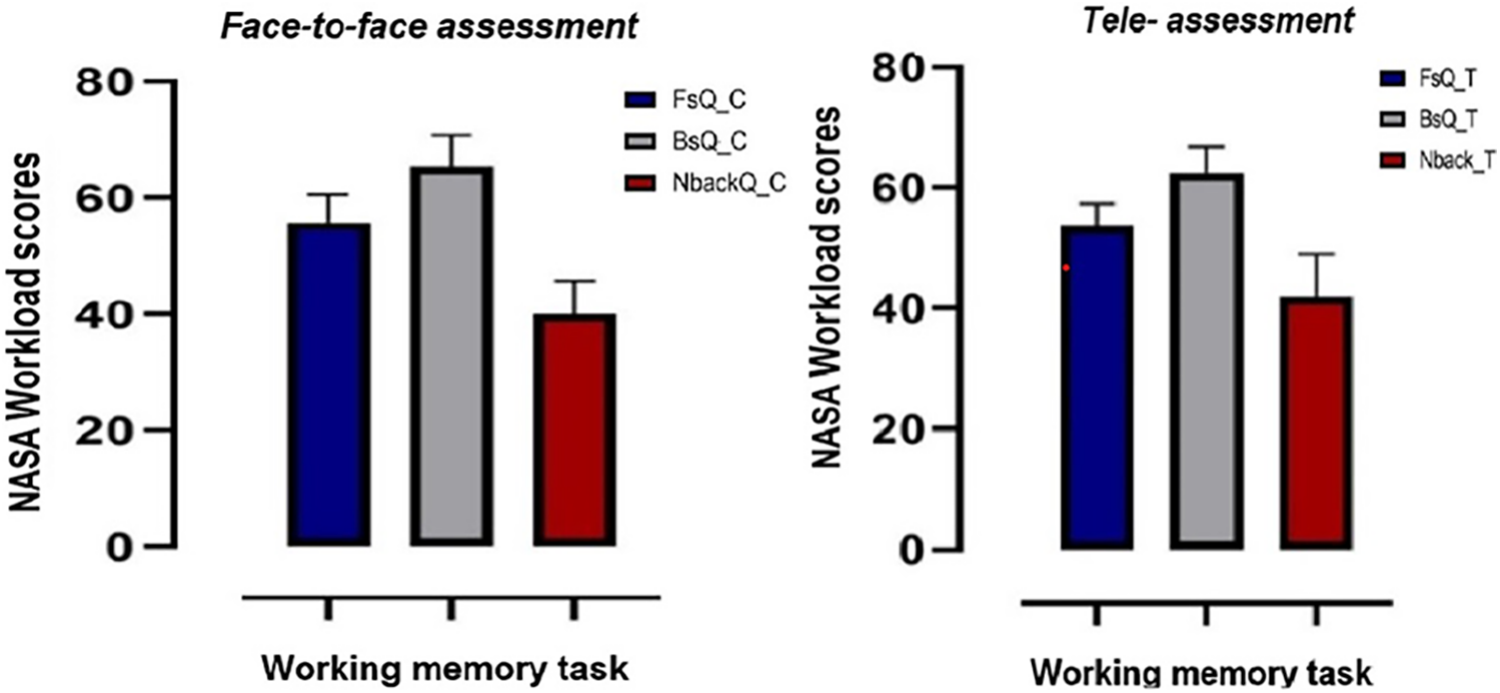
The NASA workload scores for each test in the conventional face-to-face (FsQ_C, BsQ_C, and 2backQ_C) and tele-assessments (FsQ_T, BsQ_T, and 2backQ_T). The error bars indicate one SD.

The results of the Friedman test analysis comparing the workload index scores of the tasks (Figure 5) in the two modes separately showed that the workload scores significantly differed across tests in both the face-to-face [*χ*^2^ (2) = 30, *p* < 0.001, *W* = 1] and tele [*χ*^2^ (2) = 29.5, *p* < 0.001, *W* = 1] modes. In the *post-hoc* Dunn–Bonferroni tests, it was shown that the workload scores for the backward digit task were comparatively higher than the other two tasks (*p* < 0.001), and the forward digit task scores were higher than the 2-back task scores (*p* < 0.001) in both the conventional face-to-face and tele-assessments.

### 3.4 Frequency of the participants’ ratings of the subscales

The frequency of participant's ratings of each subscale in the workload questionnaire was averaged. It was subjected to the chi-square test, which showed that a number of participants rated the backward span task as temporally demanding (*p* = 0.19), followed by mentally demanding (*p* = 0.70), in both the conventional and tele modes of assessment.

## 4 Discussion

This study compared tele-assessment to conventional face-to-face WM measures to identify any potential barriers to the implementation of virtual assessment before a larger community-based implementation of tele-assessment. Using a series of WM tasks, this study explored whether there were differences in performance in WM tasks measured through tele-assessment and face-to-face assessment in young adults with normal hearing. No statistically significant differences in performance in the forward digit span, backward digit span, and 2-back tasks were observed between the tele-assessment and face-to-face assessment (Figure 3). These results indicate that the participants performed similarly in both types of administration. The congruence between previous studies (17, 25) and our study further validates tele-assessment of WM. This study extends the findings of the earlier studies to show that despite technological limitations in connectivity, WM tasks in the auditory domain can also be administered with good reliability in tele-assessments. The fact that multiple studies have observed similar results across different cognitive tasks, domains, and assessment methods lends robustness to the argument that the mode of assessment, whether face-to-face or tele-assessment, does not significantly affect the participants’ performance. This consistency in findings across various studies highlights the potential reliability and applicability of tele-assessment methods for WM and cognitive tasks.

The findings from the NASA workload index questionnaire add significant depth to our understanding of the impact of assessment modes on participants’ perceived workload and cognitive burden. This questionnaire, which aims to measure the workload across various dimensions, was instrumental in assessing the potential differences between the face-to-face and tele modes of assessment. The results demonstrated that there were no substantial differences in the participant's subjective assessment of workload between the two assessment modes (Table 3). The NASA workload index for each mode of assessment displayed no significant differences in the ratings and weighted measures of the six scales between the two modes. The participants indicated that the WM tasks were temporally and mentally demanding (Table 1) as they required more attention and retention of auditory stimuli. However, these demands seemed to be consistent across both assessment modes. This aligns with the idea that the cognitive requirements of WM tasks, such as attention and auditory stimuli retention, transcend the assessment mode and are similarly challenging.

**Table 3:** Overall NASA workload scores for all six scales in the conventional face-to-face (FsQ_C, BsQ_C, and 2backQ_C) and tele-assessments (FsQ_T, BsQ_T, and 2backQ_T).

Subjects	FsQ_C	FsQ_T	BsQ_C	BsQ_T	2backQ_C	2backQ_T
1	56	53	69	62	39	47
2	59	53	62	64	34	39
3	60	52	71	60	48	49
4	53	53	69	60	36	31
5	60	52	65	60	48	49
6	56	53	67	62	39	47
7	53	60	69	77	33	36
8	59	56	69	65	48	47
9	60	52	71	60	48	49
10	59	53	67	62	39	47
11	56	53	67	62	39	47
12	59	53	62	64	34	39
13	43	44	51	59	39	31
14	53	57	63	60	43	39
15	50	52	59	60	34	31

Interestingly, while some participants perceived the WM tasks as temporally demanding, this perception was more pronounced in the tele-assessment (66% in tele-mode and 60% in face-to-face mode), which can be partially attributed to the technical issues, mainly interruptions in Internet connectivity, inherent to this mode. The acknowledgment of the technical challenges affecting the temporal demands underscores the importance of refining the tele-assessment process to ensure a seamless experience for participants. Furthermore, the participants reported varying levels of effort exerted and weighting of overall performance, yet these differences between the two modes of assessment were not significant. This highlights the resilience of the assessment modes in eliciting a consistent performance across participants. Overall, the subjective ratings of workload score indicate no significant differences between the face-to-face and tele modes of assessment, which shows the feasibility of tele-assessment in WM assessment for the auditory-based tasks considered in the study.

In interpreting the findings related to the mental load across WM tasks, this study draws upon the literature. Based on the participants' ratings and weighted subjective assessment using the NASA workload index, the backward digit task (BsQ) was difficult, with higher scores than the forward digit task (FsQ) and n-back task (2backQ) in the tele-assessments and in the face-to-face mode of assessment (Figure 5). These findings can be attributed to different factors including the greater attentional demands required for the backward digit span task (18) and the reversal in directionality when recalling the digits in the backward digit span task (26). Many studies have also reported a recency effect in which participants could recall the last few digits in a sequence of digits that were presented in the backward digit task more easily compared to the first few digits in the sequence (27).

The results of our pilot study show that tele-assessment of WM can be utilized as effectively as face-to-face assessments. Tele-assessments place fewer time constraints on the clinician as the software allows for automatic scoring of participant responses at the end of the test and reduces the number of visits for the participants. Although there were limitations during the tele-sessions including Internet connectivity concerns, limited professional assistance, and no direct feedback from the examiner, the participants stated that the tele-session was better than face-to-face sessions as it was more comfortable and time-saving. Given that the time gap between the face-to-face and tele-assessment measures was short (3–5 days), our results should be interpreted with caution and need further validation. Despite the short gap between the two assessment modes, the order of administration across participants was counterbalanced to minimize confounds related to repetition effects. Procedural variations, including extending the time gap between the face-to-face and tele-assessments, examining the impact of the interval between the two assessment modes on test reliability and validity, and inclusion of a larger data sample are warranted before generalization of the findings.

## 5 Conclusion

The outcome of the current study on tele-assessment of cognition in young adults with normal hearing provides insights into conducting cognitive assessment in those with hearing loss or other clinical populations and serves as a starting point for future research focused on the validation of cognition assessment through web-based platforms for both healthy populations and those impacted by detrimental effects due to aging. The congruence between how the participants perceived their cognitive task performance and the workload ratings collectively underscores the feasibility of tele-assessment. The absence of significant differences in workload and task performance suggests that tele-assessment holds promise as a viable alternative to face-to-face assessment, with its potential benefits warranting further exploration and optimization. In summary, the study suggests that tele-assessment is a valid means of virtual and remote administration of WM tests as the test scores in both conditions were consistent.

## Data Availability

The original contributions presented in the study are included in the article, further inquiries can be directed to the corresponding author.
